# Still births, neonatal deaths and neonatal near miss cases attributable to severe obstetric complications: a prospective cohort study in two referral hospitals in Uganda

**DOI:** 10.1186/s12887-015-0362-3

**Published:** 2015-04-17

**Authors:** Annettee Nakimuli, Scovia N Mbalinda, Rose C Nabirye, Othman Kakaire, Sarah Nakubulwa, Michael O Osinde, Nelson Kakande, Dan K Kaye

**Affiliations:** Department of Obstetrics and Gynecology, School of Medicine, College of Health Sciences, Makerere University, P.O. Box 7072, Kampala, Uganda; Department of Nursing, School of Health Sciences, College of Health Sciences, Makerere University, P.O. Box 7072, Kampala, Uganda; Department of Obstetrics and Gynecology, Jinja Regional Hospital, Jinja, Uganda; Clinical Operations and Health Services Research Program, Joint Clinical Research Centre, P. O. Box 10005, Kampala, Uganda

## Abstract

**Background:**

Neonatal near miss cases occur more often than neonatal deaths and could enable a more comprehensive analysis of risk factors, short-term outcomes and prognostic factors in neonates born to mothers with severe obstetric complications. The objective was to assess the incidence, presentation and perinatal outcomes of severe obstetric morbidity in two referral hospitals in Central Uganda.

**Methods:**

A prospective cohort study was conducted between March 1, 2013 and February 28, 2014, in which all newborns from cases of severe pregnancy and childbirth complications were eligible for inclusion. The obstetric conditions included obstetric haemorrhage, hypertensive disorders, obstructed labour, chorioamnionitis and pregnancy-specific complications such as malaria, anemia and premature rupture of membranes. Still births, neonatal deaths and neonatal near miss cases (defined using criteria that employed clinical features, presence of organ-system dysfunction and management provided to the newborns were compiled). Stratified and multivariate logistic regression analysis was conducted to identify risk factors for perinatal death.

**Results:**

Of the 3100 mothers, 192 (6.2%) had abortion complications. Of the remainder, there were 2142 (73.1%) deliveries, from whom the fetal outcomes were 257 (12.0%) still births, 369 (17.2%) neonatal deaths, 786 (36.7%) neonatal near misses and 730 (34.1%) were newborns with no or minimal life threatening complications. Of the 235 babies admitted to the neonatal intensive care unit (NICU), the main reasons for admission were prematurity for 64 (26.8%), birth asphyxia for 59 (23.7%), and grunting respiration for 26 (11.1%). Of the 235 babies, 38 (16.2%) died in the neonatal period, and of these, 16 died in the first 24 hours after admission. Ruptured uterus caused the highest case-specific mortality of 76.8%, and led to 16.9% of all newborn deaths. Across the four groups, there were significant differences in mean birth weight, p = 0.003.

**Conclusions:**

Antepartum hemorrhage, ruptured uterus, severe preeclampsia, eclampsia, and the syndrome of Hemolysis, Elevated Liver Enzymes, Low Platelets (HELLP syndrome), led to statistically significant attributable risk of newborn deaths (still birth or neonatal deaths). Development of severe maternal outcomes, the mothers having been referred, and gravidity of 5 or more were significantly associated with newborn deaths.

## Background

Despite global declines in infant mortality rates in the last decade, neonatal mortality rates have remained relatively unchanged in most of sub-Saharan Africa [1]. Neonatal deaths include early neonatal deaths (END or death of a baby within seven days of extra uterine life) and late neonatal deaths (deaths that occur after the first week of extrauterine life but within the first 28 days after birth). Like perinatal deaths, early neonatal deaths are largely as a result of complications of childbirth, and an estimated six million perinatal deaths occur worldwide [2]. The low- and middle-income countries carry the greatest burden of 98% of the perinatal deaths [3-5]. END accounts for three quarters of neonatal deaths and about 30 to 50% of these newborn deaths occur on the first day of life [5].

Assessment of maternal near miss morbidity, which is defined as mothers who narrowly survive death from life threatening complications of pregnancy or childbirth, is recommended as a measure of the quality of maternal health care [6-12]. A maternal near miss is defined as “a woman who, being close to death, survives a complication that occurred during pregnancy, delivery or up to 42 days after the end of her pregnancy” [6]. As a related concept, a neonatal near miss case refers to a neonate that presents with a severe life-threatening complication during the neonatal period but survives [11,12]). They include newborns with low Apgar score (less than 7 at 5 minutes), very low birth weight (less than 1,500 g) or prematurity (of 30 weeks of gestational age or less) [12] Other criteria include newborns with neonatal convulsions, septicaemia, or severe jaundice, who very often require admission to the neonatal intensive care unit [NICU].

Like for maternal near miss, three categories of criteria are used to diagnose a neonatal near miss [12-14]: The *clinical criteria include features such as* lethargy, failure to suckle, prematurity, low birth weight and hypothermia). The laboratory criteria assess e*vidence of organ-system dysfunction* (metabolic, respiratory, neurological or cardiovascular). The evidence includes severe hypoglycaemia, severe jaundice, encephalopathy, sepsis, electrolyte imbalance or thrombocytopenia). The *management-based criteria* include, among others, total parenteral feeding, tracheal intubation, continuous positive airway pressure, surgery or blood transfusion).

There is scarce data on how much maternal near miss morbidity contributes to neonatal near miss and early neonatal death. Likewise, predictors of neonatal death and prognostic factors of neonatal near miss are not well documented. Pileggi et al [11] used the following set of neonatal near miss indicators: very low birth weight (< 1,500 g), gestational age of less than 30 weeks and/or an Apgar score of less than 7 in the 5th minute of life. In this study, the early neonatal mortality rate was 8.2 deaths per 1,000 live births, while the neonatal near miss rate was 21.4 per 1000 live births, with a sensitivity of 82.6%, specificity of 97.9% and positive likelihood ratio of 37. Avenant [12] proposed a set of neonatal near miss indicators. These include respiratory failure/dysfunction (which occurred in 63% of cases), infections (which occurred in 23% of cases) and central nervous system failure/dysfunction (which occurred in 5% of cases).

The challenge of determining effective neonatal near miss indicatorsis dependent partly on absence of simple valid and reliable criteria for identification of severe neonatal morbidity, which could be used in quality improvement in health facilities or the community. Additionally, some of these conditions are rarely diagnosed. Furthermore, many indicators, particularly those of organ-system dysfunction, are not routinely registered in medical records, especially in low and middle-income countries. A prospective cohort study performed on 341 newborns with severe perinatal morbidity (secondary to severe obstetric complications) admitted to the neonatal intensive care unit found an incidence of early neonatal death of 109 deaths per 1000 live births [15]. In this study, fetal distress before birth (adjusted risk ratio [aRR] 31.29, 95% CI, 4.17–234.20, p = 0.001) and inadequate fetal heart monitoring during labor (aRR 6.0, 95% CI 1.40–25.67, p = 0.016) were significantly associated with early neonatal death [15]. The results, as well as the results of a birth survey in Brazil [16], show that obstetric complications have a significant impact on the risk of perinatal and neonatal deaths. The objectives of our study were to assess the attributable risk of still births and neonatal deaths from severe obstetric morbidity, as well as evaluate risk factors for perinatal deaths and neonatal near miss cases.

## Methods

### Study setting and design

This was a prospective cohort study of women admitted with severe obstetric complications, as well as their newborn. The study was conducted between March 1 2013 and February 28, 2014, at Mulago hospital, Uganda’s national referral hospital and the teaching hospital for Makerere University. The hospital has over 1500 beds, of which over 400 are maternity beds, and conducts over 30,000 deliveries per year. Jinja is a large regional referral hospital that serves about six district hospitals in Central and Eastern Uganda. It has a capacity of over 900 beds of which over 200 are maternity beds, and conducts over 10,000 deliveries per year.

### Data collection

Women who consented to participate were recruited in the study. Using an interviewer-administered questionnaire, and through review of medical records, data was collected on socio-demographic characteristics, obstetric history, current pregnancy complications and pregnancy outcomes up to hospital discharge. Maternal near misses were classified using the WHO criteria [6]. The neonatal near miss cases were after modification of the criteria by Pillegi et al [11] and Avenant et al [12] as follows: an Apgar score of less 3 or less at 5 minutes after birth, gestational age based on the last menstrual period less than 30 weeks, and birth weight less than 1500 g The following variables, obtained from mothers’ and newborns’ medical records, were used as possible predictors of neonatal death:maternal socio-demographic characteristics,, gestational age based on the last menstrual period, obstetric ultrasonography and obstetric and neonatal assessment; and birth weight (grouped as < 1,500 g, 1,500 g–2,499 g, and ≥ 2,500 g).

In this study***,*** an Apgar score of 7 or less at 5 minutes after birth, gestational age based on the last menstrual period less than 30 weeks, and birth weight less than 1500 g,); use of mechanical ventilation any time after birth; use of supplemental oxygen after birth; admission to the neonatal intensive care unit; blood transfusion or administration of any blood products, use of vasoactive agents, phototherapy; use of continuous positive airway pressure; intubation; cardiac massage or cardiopulmonary resuscitation; the use of phototherapy in the first 72 hours of life; use of surfactant, parenteral antibiotic administration in the first 48 hours of life; neonatal convulsions or use of anticonvulsant drugs, neonatal respiratory morbidity (transient tachypnea, hyaline membrane disease, pulmonary hypertension and meconium aspiration syndrome); hypoglycemia or necrotizing enterocolitis were used as indicators of neonatal near miss in case the babies who developed these conditions survived the neonatal period. .

### Sample size estimation

Assuming a power of 80% at the 95% significance level, a maximum accepted error of 2%, and an assumed incidence ratio of neonatal near miss of 424 per 15,169 deliveries [15], our sample size was estimated to be 279 newborns with neonatal complications.

### Data analysis

At bivariate analysis, we analyzed risk factors for severe neonatal outcomes (neonatal near miss or neonatal death). Categorical variables were compared with *X*^*2*^ square or Fisher’s exact test and continuous variables with a two-tailed student *t* test. In addition, we analyzed factors associated with risk of newborn deaths (still births and neonatal deaths) using log binomial regression analysis, where characteristics of newborn deaths (still births and neonatal deaths) and those of survivors (neonatal near miss and babies with no or minimal complications) were compared and adjusted relative risks computed. Newborns that presented with at least one of the predictors selected for multivariable analysis and survived the neonatal period were considered neonatal near miss cases. Collinear variables were evaluated using the variance inflation factor whereby the variable with the lowest p-value was selected. We conducted stratified analysis of the maternal clinical conditions associated with maternal near miss cases. For multivariate analysis using log binomial regression, variables with a p-value of less than 0.2 after the bivariate analysis, and those of importance from a clinical standpoint, were maintained in the final model, after assessing the effect of confounding and interaction. The model used assessed maternal socio-demographic characteristics adjusted for development of severe maternal outcomes, referral status and timing of development of obstetric complications.

### Ethical considerations

This research was part of a post-doctoral research project: *Evaluation and surveillance of the impact of maternal and neonatal near-miss morbidity on the health of mothers and infants*. Ethical approval to conduct the study was obtained from the Ethics and research committees of Mulago hospital, the School of Medicine, Makerere University College of Health Sciences and Uganda National Council for Science and Technology. Permission to conduct the study was obtained from the department of Obstetrics and Gynecology, Makerere University, and from the management of Mulago and Jinja hospitals. All participants gave written informed consent to be interviewed or for their newborns to be investigated.

## Results

Of the 3100 mothers, there were 192 (6.2%) women with abortion complications and 958 women (30.9%) with antepartum complications such as febrile illness, bronchial asthma, anemia and urinary tract infection. Of the remainder, there were 2142 (73.1%) deliveries. For those who delivered, the fetal outcome was 257 (12.0%) still births, 369 (17.2%) early neonatal deaths, 786 (36.7%) neonatal near misses and 730 (34.1%) were newborns with no or minimal life threatening complications. (Newborns with no or minimal complications referred to babies who survived the neonatal period but did not have any of the features used as clinical criteria, management-based criteria or organ-system dysfunction criteria for neonatal near miss).

Table 1 shows the risk of death attributable to the severe obstetric morbidity. Of the 1885 live births, 235 (18.4%) were admitted to the neonatal intensive care unit (NICU). The main reasons for admission were prematurity for 64 newborns (26.8%), birth asphyxia for 59 newborns (23.7%), and grunting respiration for 26 newborns (11.1%). The conditions associated with severe obstetric morbidity contributed to 242 of the 257 still births (94.1%) and 196 of the 369 neonatal deaths (53.1%). Of the 235 babies admitted to the NICU, 38 (16.2%) died in the neonatal period, and of these, 16 died in the first 24 hours after admission. Ruptured uterus caused the highest case-specific mortality of 76.8%, and led to 16.9% of all newborn deaths. Regarding specific causes of newborn deaths, the commonest cause was severe birth asphyxia associated with severe obstructed labor for 120 newborns (19.2%). This was followed by obstructed labor (110 newborns, 17.6%), ruptured uterus (106 newborns, 17.3%) and eclampsia/severe preeclampsia (18 cases, 2.9%).

**Table 1:** **Attributable risk (case-specific mortality) of newborn deaths from the severe obstetric morbidity**

**Obstetric complication**	*Number of Still births (n = 257)	*Number of Neonatal deaths (n = 369)	Ω**βPercentage of the newborns deaths related to the condition**	**∞Percentage of all newborn deaths**
*Hypertensive disorders*				
Severe preeclampsia (n = 218)	20	63	38.1	13.3
Eclampsia (n = 172)	32	28	34.9	9.6
Chronic hypertension with superimposed				
Preeclampsia (n = 4)	2	1	75	0.5
HELLP syndrome (n = 9)	5	1	66.7	1.0
*Obstetric hemorrhage*				
& Hemorrhage from late abortion (n = 38)	5	5	26.3	1.6
Antepartum hemorrhage (n = 116)				
Ruptured uterus (n = 138)	32	28	51.7	9.6
	82	24	76.8	16.9
Obstructed labor (n = 310)	64	46	35.5	17.6

& These were abortions at gestation age greater than 20 weeks.

*The remainder of still births and neonatal deaths were from other causes unrelated to obstetric complications of childbirth.

ΩTotal number of newborns deaths divided by the total number of women with the obstetric condition.

∞Total number of newborn deaths in the stratum divided by the total number of newborn deaths; β Newborn deaths include still births and neonatal deaths.

Table 2 shows the socio-demographic characteristics and medical histories of the mothers of the newborns. The four groups (still births, neonatal deaths, near misses, and babies with minimal complications) differed significantly with regard to gravidity, parity, age category, marital status, education level, and employment status. Likewise, the four groups differed significantly (p < 0.05) regarding referral status, and timing of the pregnancy complications, development of severe maternal outcomes in the mothers, admission of the mothers to the high dependency obstetric unit (or the intensive care unit, and the mode of delivery. The overall mean birth weight was 3057 ± 357 g, and 338 (15.8%) babies had low birth weight, while 174 (8.1%) had weight exceeding 4000 g. The mean birth weight of the still births was 2978 ± 980 g, while that of neonatal deaths was 2647 ± 906 g. While the mean birth weight of the neonatal near miss was 3307 ± 1708 g, the mean birth weight of newborns with no complications was 3330 ± 1060 g. Therefore, across the four groups (still births, neonatal deaths, neonatal near miss cases and newborns with minimal or no complications), there were significant differences in mean birth weight, p = 0.003.

**Table 2:** **Socio-demographic characteristic and medical histories of the mothers of newborns displayed according to neonatal outcomes**

**Characteristics**	**All newborns (n = 2142) N (%)**	**Stillbirths (n = 257) N (%)**	**Neonatal deaths (n = 369) N (%)**	**Neonatal near miss (n = 786) N (%)**	**No complications (n = 730) N (%)**	**p-value (testing difference in groups)**
*Age category*						
18 years or less	183 (8.5)	19 (7.4)	34 (9.2)	52 (6.7)	78 (10.7)	<0.001
19–24 years	853 (39.8)	77 (30.0)	144 (39.0)	307 (39.1)	325 (44.5)	
>24 years	1106 (51.7)	161 (62.6)	191 (51.8)	427 (54.3)	327 (44.8)	
*Gravidity*						
1	702 (32.8)	50 (31.1)	117 (31.7)	205 (26.1)	330 (45.2)	<0.001
2–4	1051 (49.1)	126 (49.0)	174 (47.2)	450 (57.3)	301 (41.2)	
5 and more	389 (18.0)	81 (19.9)	78 (21.0)	131 (16.6)	99 (13.6)	
*Marital status*						
Single	378 (17.6)	32 (12.5)	80 (21.7)	152 (19.3)	116 (15.9)	0.015
Married	1764 (82.4)	226 (87.5)	289 (78.3)	633 (80.7)	613 (84.1)	
*Employment status*						0.006
Formal	210 (9.8)	21 (8.2)	29 (7.9)	97 (12.3)	63 (8.6)	
Informal	682 (31.8)	90 (35.0)	125 (33.9)	237 (30.2)	230 (31.5)	
Unemployed	1247 (58.4)	146 (56.8)	214 (58.2)	452 (57.5)	435 (59.9)	
*ΩEducation level*						
None or primary						0.002
Secondary	872 (40.7)	129 (50.2)	154 (41.7)	321 (40.8)	268 (36.7)	
Post-secondary						
	1022 (47.7)	112 (43.5)	178 (48.2)	371 (47.2)	361 (49.5)	
	233 (11.6)	16 (6.3)	34 (10.1)	85 (10.8)	98 (13.4)	
*Referral status*						
Referred	1418 (66.2)	189 (73.5)	257 (69.6)	512 (65.1)	60 (8.2)	0.009
*Not referred	724 (33.8)	68 (26.5)	112 (31.4)	276 (34.9)	670 (91.8)	
*Timing of complications*						
Occurred before admission	1159 (54.1)	94 (36.6)	202 (54.7)	676 (86.0)	187 (25.6)	0.001
Occurred before arrival and new complications developed	540 (25.2)	115 (44.7)	95 (25.7)	73 (9.3)	257 (35.1)	
Complications occurred during hospitalization	443 (20.7)	48 (18.7)	72 (19.5)	37 (4.7)	286 (39.3)	
*Severe maternal outcomes*						<0.001
Present	1444 (67.4)	79 (30.7)	154 (41.7)	671 (85.3)	540 (74.0)	
Absent	698 (32.6)	178 (69.3)	215 (58.3)	115 (14.7)	190 (26.0)	
*Admission to HDU*						0.001
Yes	530 (32.9)	154 (59.9)	146 (39.6)	72 (9.2)	158 (21.6)	
No	1612 (67.1)	103 (40.1)	223 (40.4)	714 (90.8)	572 (78.4)	
Mode of delivery						
Vaginal	926 (43.2)	169 (45.8)	123 (33.3)	642 (81.7)	624 (85.5)	0.005
ΩAbdominal	1207 (56.3)	197 (53.4)	243 (65.9)	143 (18.3)	103 (14.1)	
Assisted delivery	9 (0.5)	3 (0.8)	3 (0.8)	0 (0.0)	3 (0.4)	

ΩCaesarean delivery or laparotomy for ruptured uterus; *Not referred include self-referrals.

Table 3 shows the risk factors for newborn deaths using the clinical criteria. Antepartum hemorrhage, ruptured uterus, severe preeclampsia, eclampsia, and HELLP syndrome led to statistically significant attributable risk of newborn deaths (still birth or neonatal deaths) (p <0.05). There was a significant decrease in newborn deaths with increasing birth weight (RR 0.44, 95% CI 0.37–0.52; p-value <0.001). However, when the weight was categorized in 3 strata of low birth weight (<2500 g), normal weight and macrosomia (>3999 g), the risk of newborn deaths was 33.26 (95% CI 21.67–50.84); p-value < 0.001 for low birth weight babies, while it was 2.85 (95% CI 2.25–3.62); p-value <0.001, for babies of birth weight greater than 3999 g.

**Table 3:** **Clinical conditions and risk of perinatal death in newborns whose mothers had severe obstetric complications**

**Characteristic**	**Perinatal and neonatal deaths (n = 626)**	Ω**Near miss and uncomplicated newborns (n = 1516)**	**Crude risk ratios and 95% CI**	**p-values**
*Hemorrhage*				
None	10 (4.1)	28 (11.6)	*Ref*	
Antepartum	60 (24.5)	56 (23.2)	3.00 (1.33–6.73)	0.008
Postpartum	69 (28.3	125 (51.9)	1.55 (0.70–3.37)	0.274
Ruptured uterus	106 (43.3)	32 (13.3)	9.07 (4.27–21.12)	<0.001
Obstructed labor	110 (17.5)	200 (13.1)	2.26 (1.56–3.62)	0.002
*Hypertensive disorders*				
Mild or no preeclampsia	461 (73.6)	1294 (85.4)	*Ref*	
Severe preeclampsia	83 (13.3)	126 (8.3)	1.84 (1.37–2.49)	0.001
Eclampsia	73 (11.6)	92 (6.1)	2.22 (1.60–3.08)	0.01
Chronic Hypertension	3 (0.5)	1 (0.0)	8.42 (0.87–81.15)	0.065
HELLP Syndrome	6 (1.0)	3 (0.2)	5.61 (1.40–22.45)	0.015

ΩNeonatal near miss cases and newborns with minimal or no neonatal complications.

Table 4 shows the risk factors for perinatal death. Grunting respiration, tracheal intubation during neonatal care, parenteral feeding, transfusion, use of surfactant, neonatal jaundice, phototherapy, use of anticonvulsants, and use of vasoactive agents accurately predicted the outcome of neonatal deaths, and were eliminated from the multivariate analysis due to collinearity. Development of severe maternal outcomes, the mothers having been referred, and gravidity of 5 or more were significantly associated with newborn deaths. Having attained a post-secondary level of education was associated with a 56% reduced risk of neonatal death (p = 0.021).

**Table 4:** **Independent risk factors for newborn deaths (still births plus neonatal deaths) for newborns whose mothers had obstetric complications**

**Characteristic**	**Still births and neonatal deaths (n = 626)**	**Near miss and **Ω**others (n = 1516)**	**Crude risk ratios and 95% CI**	**p-values**	**Adjusted risk ratios and 95% CI**	**p-values**
*∞Severe maternal outcome*						
Yes	393 (62.8)	305 (20.1)	6.70 (5.45–8.22)	<0.001	2.87 (4.52–7.19)	<0.001
No	233 (37.2)	1211 (79.9)	*Ref*		*Ref*	
*Age category*						
18 years or less	53 (8.5)	130 (8.6)	*Ref*		*Ref*	
19–24 years	221 (35.3)	632 (41.7)	0.85 (0.60–1.22)	0.396	0.93 (0.60–1.45)	0.747
>24 years	352 (56.2)	754 (49.7)	1.14 (0.81–1.61)	0.440	1.04 (0.64–1.70)	0.874
*Gravidity*						
1	167 (26.7)	535 (35.3)	*Ref*		*Ref*	
2–4	300 (47.9)	751 (49.5)	1.29 (1.03–1.58)	0.028	1.31(0.97–1.76)	0.074
5 and more	159 (25.4)	230 (15.2)	2.21 (1.70–2.89)	<0.001	2.06 (1.36–3.12)	0.001
*Employment status*						
Formal	50 (8.0)	160 (10.6)	*Ref*		*Ref*	
Informal	215 (34.3)	467 (30.8)	1.47 (1.03–2.10)	0.033	0.99 (0.60–1.60)	0.832
Unemployed	360 (57.5)	887 (58.6)	1.30 (0.92–1.82)	0.132	0.97 (0.60–1.55)	0.931
*Ω* *Education level*						
None or primary l	283 (45.7)	589 (38.9)	*Ref*		*Ref*	
Secondary level		732 (48.3)	0.82 (0.68–1.02)	0.054	0.92 (0.71–1.18)	0.548
Post-secondary	290 (46.3)	183 (12.1)	0.48 (0.41–0.55)	<0.001	0.56 (0.34–0.90)	0.021
	50 (8.0)					
*Referral status*				0.002		
Referred	446 (71.2)	972 (64.1)	0.72 (0.59–0.88)		0.51–0.85	0.002
Not Referred	180 (28.8)	544 (35.9)	*Ref*		*Ref*	
*Timing of complications*						
Occurred before admission	296 (47.3)	863 (56.9)	*Ref*		*Ref*	
£New complications developed	210 (33.5)	330 (21.8)	1.12 (1.00–1.26)	<0.001	0. 95 (0.72–1.25)	0.719
Complications occurred during hospitalization						
	120 (19.2)	323 (21.3)	0.80 (0.45–1.07)	0.074	0.76 (0.56–1.02)	0.070

∞Severe maternal outcomes include maternal deaths and maternal near miss cases.

ΩNeonatal near miss cases and newborns with minimal or no neonatal complications.

*CI* Confidence intervals; £New complications occurred after admission.

## Discussion

The study shows that severe obstetric complications contribute significantly to newborn deaths (still births and neonatal deaths) and neonatal near miss cases. Therefore, neonates of mothers with severe obstetric complications represent a sub-group of neonates at very high risk of morbidity and mortality. The findings are in agreement with the global literature that birth asphyxia and prematurity attributable to severe obstetric complications are the most important cause of still births and neonatal deaths [5,17-19]. Overall, 16.2% of neonates admitted to the NICU died in the neonatal period, with the majority dying in the first 24 hours after admission.

The challenge of using neonatal near miss indicators is partly due to absence of simple valid and reliable criteria for identification of severe neonatal morbidity, which could be used in quality improvement in health facilities [20]. Pillegi-Castro et al [20] developed a criteria of pragmatic markers (birthweight <1750 g, Apgar score at 5 minutes <7, and gestational age <33 weeks), which were validated using data from the World Health Organization Global Survey on maternal and perinatal health, and the WHO Multicountry Survey on Maternal and Newborn Health (WHOMCS). The diagnostic accuracy of the pragmatic and management markers of severity for identification of early neonatal deaths [20] showed high sensitivity, 92.8% (95% CI 91.8–93.7%), specificity, 92.7% (95% CI 92.6–92.8%), positive likelihood ratio, 12.7 (95% CI 12.5–12.9); negative likelihood ratio, 0.08 (95% CI 0.07–0.09); with a diagnostic odds ratio, 163.4 (95% CI 141.6–188.4).

These results of high neonatal mortality are similar to that of an earlier study [15] which showed an early neonatal death of 10.9%, as well as results of a study at a regional referral hospital in Sudan where also only inborn neonates were included [21]. The results are also similar to those of a study on newborns admitted to the NICU in Tanzania in 2003 [22], which reported a 19% neonatal mortality, as well as those of a study from Nigeria [23] which showed a neonatal mortality rate of 20.3%.

The finding that birth asphyxia is a major cause of neonatal death is consistent with earlier studies at the hospital [16,24] as well as results of a study at a university hospital in Tanzania [22]. One explanation of the high number of deaths due to asphyxia in our data may be the definition criteria for asphyxia that we used (fetal bradycardia or a 5-minute Apgar score less than 4). This may have included fetal distress in preterm babies, where the asphyxia may be attributable to prematurity per se, from respiratory distress syndrome or transient tachypnea of the newborn. Indeed, prematurity was a major indication for admission to the NICU in our study. Previous studies have shown prematurity to be a major cause of neonatal death [19-23].

As a limitation, we acknowledge that details of the cases of complications of childbirth are limited to the intrapartum and the severe obstetric complications that eventually led to childbirth. For other obstetric but not child-birth related complications, the data was not available. Despite this, our findings have major implications for intrapartum care, particularly provision of essential newborn care. Improved monitoring of labor, basic training on newborn resuscitation skills and proper newborn resuscitation immediately after birth may be critical in reducing neonatal mortality as well as still births, as some babies may be wrongly classified as still birth in situations where few or no attempts are made at new born resuscitation of severely asphyxiated newborns. Previous studies have shown than mortality among babies born with birth asphyxia may be as high as 40% [25-28]. While a study in six developing countries [29] showed that training on Essential Newborn Care (including training on basic resuscitation) had no effect on early neonatal mortality, it revealed significant reduction in the rate of fresh stillbirths primarily fresh. This was most likely as an effect of improved resuscitation of asphyxiated babies who would have been misclassified as stillbirths prior to the training [29].

## Conclusion

Antepartum hemorrhage, ruptured uterus, severe preeclampsia, eclampsia, and HELLP syndrome led to statistically significant attributable risk of newborn deaths (still birth or neonatal deaths). Development of severe maternal outcomes, the mothers having been referred, and gravidity of 5 or more were significantly associated with newborn deaths.
